# News and Events of the Week

**Published:** 1911-05-13

**Authors:** 


### News and Events of the Week.

The Council of the University of Lyons decided recently to create a chair of experimental surgery, and to this Dr. Villard, surgeon of the hospitals, has now been appointed.

In the Sandwich Islands great success seems to have attended the use of calcium carbide for the destruction of the larvæ of mosquitoes. It is said to be much more effective than oil and almost as inexpensive.

The Belgium medical inspectors of school-children have founded a national association to co-operate with the existing international association of medical inspectors of schools, to institute international congresses, and to defend the interests of its members.

Two further names must be added to the roll of those who have given their lives in attempting to combat the plague in Manchuria, those of Elie Vassilievitch Mamontoff, a student of the Military Academy of Medicine of St. Petersburg, and John Broujoumas, an assistant physician.

A Bill for the registration of masseurs and masseuses in New South Wales will be introduced during the next session of the State Parliament. Its object is to protect the public by insisting on a thorough eighteen months' course of training, including teaching in anatomy, physiology, medical electricity, etc.

A sum of £600 a year for ten years has been offered to the University of Tasmania by the trustees of the estate of Mr. John Ralston to found a chair of biology. The incumbent is to devote his time to research work in the diseases of plants and animals in Tasmania, in the anatomy and development of marsupials and in bacteriology. The first professor is to be Mr. T. T. Flynn, at present the university lecturer in biology.

Lady Juliet Duff has received the following further contributions to free Charing Cross Hospital from its mortgage debt: Bazaar at St. Helen's School, Streatham, £48 6s.; anonymous, £26 5s.; Miss Matilda Levy, £26 5s.; proceeds of lecture organised by Dr. Naesmyth, £12 18s.; Mr. W. Heward Bell and Mr. Carl Schelling, £10 10s. each; Mrs. Pink, Mrs. George Mosenthal, the Savage Club, and Messrs. Dewrance and Company, £5 5s. each.

Dr. Nathaniel H. Alcock, Vice-Dean and Lecturer in Physiology at St. Mary's Hospital Medical School, London, has been appointed to the Chair of Physiology at McGill University, Montreal. The new Professor took his B.A. degree at Trinity College, Dublin, in 1893, being senior medallist, and proceeded to the M.D. in 1896. In 1909 he obtained the D.Sc. of London University. He is joint author with Dr. Ellison of a text-book of elementary physiology.

General Medical Council.—Mr. M. A. Curry, late medical officer at Germiston, Transvaal, who served as a civil surgeon with the Guards Brigade; Dr. A. Hawkyard, president of the Leeds and District Medical Practitioners' Association; Mr. G. Jackson, F.R.C.S., of Plymouth, an ex-president of the Incorporated Medical Practitioners' Association; and Dr. J. A. MacDonald, of Taunton, chairman of the special Poor Law Committee of the British Medical Association, are candidates for election as a direct representative on the General Medical Council to succeed Dr. L. S. McManus, of Battersea.

A lecture on "Food Adulteration and Food Substitutes," their effect on health, will be given by Professor Halliburton at 34 Devonshire Street, Harley Street, London, W., on Tuesday, May 16, at 5 p.m., under the auspices of the Institute of Hygiene.

Speaking at the annual meeting of the Christian Literature Society for China, held at the Queen's Hall on Tuesday (May 3), Surgeon-General Evatt presiding, the Secretary of the China Emergency Committee (Rev. E. T. Reed) said the Church ought to be most devoutly thankful that, in connection with the medical profession, the medical missionaries of China had really laid the foundation for medical and surgical science in that vast empire. They had translated numerous medical works into Chinese, and he supposed the medical men of China of the future would find their chief mental sustenance in the work which had already been done by the medical missionaries.

